# Phage Displayed Peptides to Avian H5N1 Virus Distinguished the Virus from Other Viruses

**DOI:** 10.1371/journal.pone.0023058

**Published:** 2011-08-22

**Authors:** Dan Wu, Guangxing Li, Chengfeng Qin, Xiaofeng Ren

**Affiliations:** 1 Department of Preventive Veterinary Medicine, College of Veterinary Medicine, Northeast Agricultural University, Harbin, China; 2 Department of Basic Veterinary Medicine, College of Veterinary Medicine, Northeast Agricultural University, Harbin, China; 3 State Key Laboratory of Pathogen and Biosecurity, Beijing Institute of Microbiology and Epidemiology, Beijing, China; University of Cambridge, United Kingdom

## Abstract

The purpose of the current study was to identify potential ligands and develop a novel diagnostic test to highly pathogenic avian influenza A virus (HPAI), subtype H5N1 viruses using phage display technology. The H5N1 viruses were used as an immobilized target in a biopanning process using a 12-mer phage display random peptide library. After five rounds of panning, three phages expressing peptides HAWDPIPARDPF, AAWHLIVALAPN or ATSHLHVRLPSK had a specific binding activity to H5N1 viruses were isolated. Putative binding motifs to H5N1 viruses were identified by DNA sequencing. In terms of the minimum quantity of viruses, the phage-based ELISA was better than antiserum-based ELISA and a manual, semi-quantitative endpoint RT-PCR for detecting H5N1 viruses. More importantly, the selected phages bearing the specific peptides to H5N1 viruses were capable of differentiating this virus from other avian viruses in enzyme-linked immunosorbent assays.

## Introduction

Since the first evidence regarding direct transmission of highly pathogenic avian influenza A virus (HPAI), subtype H5N1 from poultry to human in 1997 and resulted in the death of 6 of the 18 infected individuals [1]–[3]. The HPAI H5N1 has become one of the most important public health concerns worldwide. At present, the virus has spread to many countries in Europe, Asia and Africa [4]. In 2009, an identified fatal influenza (H5N1) infection in a human was reported on January 17, 2009 [5]. Increased geographical distribution and continued evolution of H5N1 viruses as well as an immunologically naïve human population has maintained the pandemic potential of these viruses [6]–[8].

In addition to vaccination and administration of antiviral drugs against H5N1 viruses, development of effective detection approaches is required to manage and control the deadly disease. Phage display is a recently developed technology and phage random peptide library consists of a pool of billions of heterologous peptides that can be produced by the fusion of random nucleic acid sequences to the N terminus of one of the capsid protein genes of a filamentous bacteriophage [9]. Phage display peptide library is a powerful tool to identify specific ligands of a target protein by a biopanning process. This technology has been applied successfully in numerous aspects, including antibody engineering [10], peptide and protein drug discovery and manufacture [11], diagnostic analysis [12] and vaccine development [13]. Herein we identified three phage clones that specifically binding to the HAPI H5N1 viruses using a 12-mer random phage library. The binding peptides of the phages were sequenced. More importantly, these identified phages were able to distinguish HAPI H5N1 from other avian viruses.

## Materials and Methods

### Cell and virus

Madin-Darby canine kidney (MDCK) cells (ATCC, Manassas, VA) were grown in Dulbecco's MEM with 1 mM l-glutamine and 10% fetal bovine serum at 37°C and 5% CO_2_ in air. HPAI H5N1 strain A/goose/Jilin/hb/2003 were propagated in the MDCK cells in the absence of serum and purified by differential centrifugation conventionally. The concentration of the purified viruses diluted in PBS was measured by Thermo Scientific NANODROP 2000 Spectrophotometer ((NanoDrop Technologies, Thermo Fisher Scientific, Wilmington, DE) and calculated by the molar absorbance coefficient A_260_/A_280_ according to the manufacturer's instructions.

### Biopanning and enrichment analysis

Phage display was done according to the manufacturer's instructions (New England Biolabs) with minor modifications. For the first round of panning, 96-well plates were coated with the H5N1 viruses at a concentration of 14 µg/well in 0.1 M NaHCO_3_ (pH 8.6) buffer overnight at 4°C. The next day, the plates were blocked for 1 h at 4°C with 5% skimmed milk diluted in 0.05% (vol/vol) Tween 20 in phosphate-buffered saline (PBST). Following six washes with TBST (50 mM Tris-HCl, pH 7.5, 150 mM NaCl, 0.1%[vol/vol] Tween 20), the viruses were incubated with the phage library at a final concentration of 2×10^11^ (100 µl/well) at room temperature for 30 min with gently rocking. Subsequently, unbound phages were removed by 10 times wash with TBST and the bound phages were eluted by adding 100 µL elution buffer (0.2 M glycine-HCl [pH 2.2]) at room temperature for 30 min. The eluate neutralized with 15 µL 1 M Tris-HCl (pH 9.1) was collected and tittered. The phages were amplified in *Escherichia coli* ER2738 and purified by polyethylene glycol precipitation.

The second and third rounds of panning were repeated under similar panning conditions in addition to the increased concentration of Tween 20 (0.5% [vol/vol]) in TBST. In the fourth round of panning, the coated viruses were replaced by the supernatant form MDCK culture. After incubation of the phages to the supernatant at room temperature for 30 min, the resulting phages were subjected to the fifth round of panning. The titer of the phages in input, elute buffer (output) and that after amplification in *E.coli* were determined to evaluate the enrichment efficiency.

### Analysis of binding of individual phage to H5N1 viruses

Positive phage clones were identified by indirect ELISA. Briefly, ELISA plates were coated with H5N1 viruses diluted in 0.1 M NaHCO_3_ (pH8.6) at a concentration of 10 µg/well. Seven controls were set: phage library coating group; phage-free group; secondary antibody-free group; porcine transmissible gastroenteritis virus coating group; avian infectious bronchitis virus coating group; blocking buffer group and virus dilution solution coating group. The coating process was done overnight at 4°C. The next day, the plates were blocked with 1% bovine serum albumin (BSA) in TBS buffer (TBSB) for 2 h at room temperature. The plates were washed six times with TBST and then incubated with individual phage from the last round of biopanning at a concentration of 2×10^11^ in 0.1 M NaHCO_3_ (pH 8.6) for 1 h at 37°C. After six washes with TBST, the M13 polyclonal antibody (dilution 1∶1,000 in TBSB; Abcam) was added to these wells for 1 h at 37°C. After six washes with TBST, the wells were incubated with the horseradish peroxidase (HRP)-conjugated anti-rabbit IgG antibody (dilution 1∶5,000 in TBSB, Sigma). The color was developed using *o*-phenylenediamine (OPD), and the optical density (OD) value was read using an ELISA plate reader at a wavelength of 405 nm. The experiments were performed in triplicate.

### PCR amplifying genes encoding the exogenous peptides of phages

Ten positive phage clones were amplified and precipitated with polyethylene glycol-NaCl. Each phage clone DNA was purified using a plasmid extraction kit (Qiagen, Germany). The purified DNA template, sense primers: 5′-TCACCTCGAAAGCAAGCTGA and antisense primer: 5′-CCCTCATAGTTAGCGTAACG were used to PCR amplify the gene encoding the exogenous peptides of M13. The PCR profile included 95°C for 5 min, 30 cycles of 95°C for 30 s, 57°C for 30 s, 72°C for 30 s. There was a final extension of 72°C for 7 min. The corresponding amino acid sequences were deduced, based on subsequent DNA sequencing.

### Sensitivity comparison among antibody-mediated ELISA, phage-mediated ELISA and RT-PCR

The sensitivity of phage–based detection was compared with antibody-based ELISA and reverse transcription (RT)-PCR to determine the minimum quantity of the virus detected. For phage-based ELISA, the H5N1 viruses serially diluted in DMEM were coated into ELISA plates overnight at 4°C. The next day, the wells were blocked with 5% skimmed milk for 3 h at room temperature. Then the selected phages and phage complex from the phage display library (control phage) diluted in PBS at a final concentration of 1.5×10^12^ was used as primary antibody. After triple washes with TBST, the wells were incubated with anti-M13 antibody (1∶1600 dilution in PBS) for 1 h followed by another incubation with HRP-conjugated goat anti-rabbit antibody (1∶5000 dilution in PBS) for 1 h. The OD_405_ value of detected phage wells (P)/negative control (N)>2 was judged as positive results. For conventional ELISA, the H5N1 viruses were serially diluted in DMEM medium and coated into ELISA plates as above. Rabbit antiserum against H5N1 viruses was serially diluted in PBS buffer and incubated with the coated H5N1 viruses for 1 h. After triple washes with TBST, the wells were incubated with HRP-conjugated secondary antibody (1∶5000 dilution in PBS) for another 1 h. The normal rabbit serum was used as negative control for ELISA and the OD_450_ of detection well (P)/that of control well (N)>2 was judged as positive results.

A conventional RT-PCR amplifying partial hemagglutinin (HA) gene (561 bp in length) of H5N1 virus was performed. The H5N1 viruses were diluted in PBS buffer and the final concentration of the viruses was adjusted to 0.647 mg/ml. Firstly, 3 ml of the viruses were subjected to viral RNA extraction using a RNA extraction kit (Fastgene, China) according to the manufacturer's instructions. The extracted viral RNA was dissolved in diethyl procarbonate treated sterile water in a volume of 40 µl. The reverse transcription system included 5 µl of viral RNA (1 µg), 7.5 µl of sterile water, 1 µl of M-MLV reverse transcriptase (TaKaRa, China), 0.5 µl of RNase Inhibitor (40 U/ml), 1 µl of Oligo dT, 1 µl of dNTP Mixture (10 mM) and 5×MLV Buffer (4 µl). The reaction was performed at 42°C for 30 min, 99°C for 5 min and 5°C for 5 min. The resulting cDNA was serially diluted and subjected to PCR. The PCR mixture included cDNA (2.5 µl), 10×Easy Taq polymerase (0.3 µl. KeyGen, China), 2 µl of dNTP Mixture (2.5 mM), 10×PCR Buffer (2.5 µl), 0.5 µl of sense primer (P1: 5′-GATACGCTGCAGACAAAGAA) and antisense primer (P2: 5′-TTCTGCATTGTAACGATCCA), and sterile water (16.7 µl). PCR parameters were composed of 94°C for 5 min, 30 cycles of 94°C for 30 s, 52.3°C for 30 s and 72°C for 40 s followed by 72°C for 10 min. The authenticity of the PCR product was confirmed by sequencing.

### Utility of the phages bearing specific peptides for viral diagnosis

The specificities of the selected phages were evaluated by using them as diagnostic reagents to detect a panel of avian viruses. These viruses included duck plague virus (DPV) a vaccine strain, avian bronchitis virus (IBV) strain Beaudette, fowlpox virus (FPV) isolate HH2008, avian infectious bursal disease virus (IBDV) strain UK661, avian infectious laryngotracheitis virus (AILV) strain K317 and newcastle disease virus (NDV) strain La Sota. The viruses were diluted in 0.1 M NaHCO_3_ (pH8.6) to a final concentration of 15 µg/well and coated onto ELISA plates overnight at 4°C. Subsequent ELISA steps were performed as above-mentioned protocols. The OD_405_ values were recorded. At least three independent experiments were carried out. Each data point was presented as mean ± SD. Statistical significance was evaluated using the t-test. “*” means a value of P<0.01 was considered statistically highly significant. Furthermore, the identified phages were used to detect an H9N2 avian influenza virus coated in ELISA plates to further analyze the specificity of the phage-based ELISA using the above-mentioned procedure.

## Results and Discussion

### Requirement of development of diagnostic agents to influenza viruses

At present, antiviral drugs are available in fighting influenza, such as the M2 inhibitors and the neuraminidase inhibitors. Nonetheless, the emergence of drug resistant influenza strains raises concern over their effectiveness. It has been reported that M2 inhibitors resistant H5N1 viruses are widespread [14]. The efficacy of the neuraminidase inhibitor, oseltamivir, appears to be very time dependant, where treatment started later than 24 hours post infection is much less effective [15]. Therefore other alternative antiviral drugs are required to fight H5N1 influenza. In vaccination, it is well known that influenza viruses are dynamic and are continuously evolving. Influenza type A viruses undergo antigenic drift and antigenic shift, resulting in new virus strains that may not be recognized by antibodies to earlier influenza strains. Therefore, rational design of vaccines against influenza vaccines still has a long way to go. Although there are difficulties of tackling influenza virus with drugs or vaccines, they are very useful in the prevention and therapy of influenza. At the same time, effective diagnostic tests for viruses screening prior to application of drugs and vaccines are widely accepted, due to their simplicity, rapidity and applicability.

### Identification of phages bearing specific peptides to H5N1 viruses

**Figure 1:**
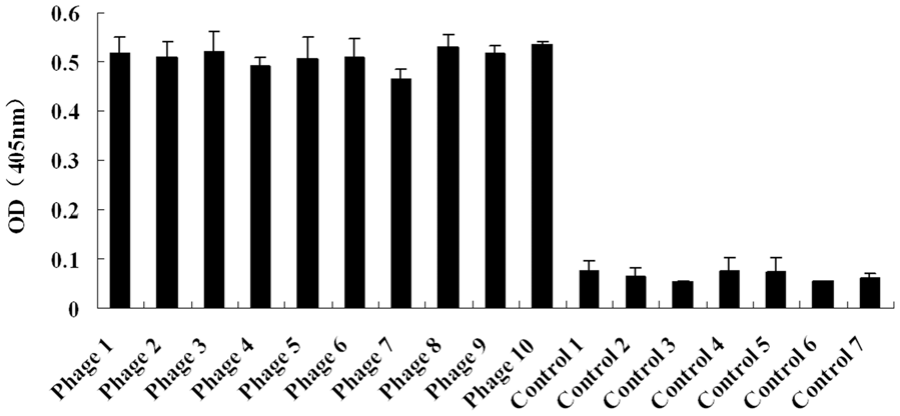
Analysis of binding of selected phages to H5N1 virues by ELISA. Ten selected phages named phages 1 to 10 were incubated with the H5N1 viruses in ELISA plates to test their binding activities to the viruses as described in Materials and methods. The experiment was performed in triplicate. The individual phage and controls are indicated in the x axis. The controls 1–6 are phage library coating group, phage-free group, secondary antibody-free group, porcine transmissible gastroenteritis virus coating group, avian infectious bronchitis virus coating group, blocking buffer group and virus dilution solution coating group, respectively. The OD_405_ values of tested individual phage and the OD value of the control is shown on the y axis.

One purpose of the current study was to use phage display to identify specific ligands of H5N1 viruses. There are reports regarding the phage-displayed peptides selected from combinatorial libraries that interacting with hepatitis B virus, adenovirus type 2, Andes virus, Sin Nombre virus and Hantaan virus and coronavirus [16]. At present, the phage display technology has become an increasingly attractive molecular tool to researchers in biotechnology related fields [17]–[19]. In our study, we used the H5N1 virion as an immobilized target and performed a biopanning using a 12-mer phage display peptide library. Since the viruses were harvested from the cells, therefore, we made a subtract panning by including the cell supernatant as a target to incubate with the selected phage to decrease the reaction background; in addition, we decrease the concentration of the viruses gradually to improve the specificity of the identified phages. Our results showed that the titer of the eluted phages was increased at the last round of panning (data not shown). Using ELISA, ten phage clones that specific binding to H5N1 viruses were identified. No positive results were found in the control, confirming the binding specificity of the phages to the H5N1 viruses (Figure 1).

### Deduced amino acids that were responsible for binding activity

**Figure 2:**
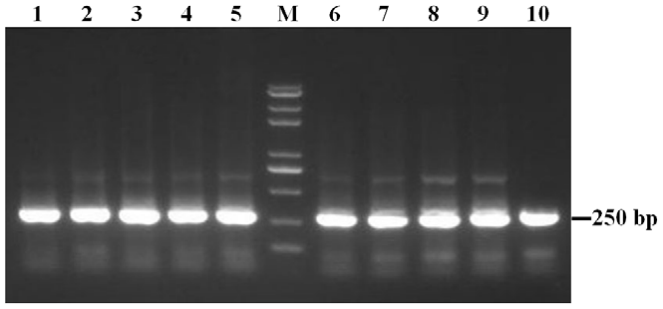
PCR amplifying genes encoding heterologous peptides in the recombinant phages. Using specific M13 phage primers, the genes encoding the heterologous peptides in the ten recombinant phages were amplified by PCR. The gel agarose electrophoresis of PCR product is provided. The PCR product from each phage clone is numbered 1 to 10 and lane M is DNA marker.

Following extraction of DNA of the selected phages, the genes encoding the peptides expressed on the surfaces of the recombinant phages were amplified by PCR. The PCR products were approx. 250 bp in size as expected (Figure 2). DNA sequencing indicated that among the 10 selected phages, three deduced peptide sequences (12 amino acids in length) were identified (Table 1). Phages bearing peptide HAWDPIPARDPF, AAWHLIVALAPN or ATSHLHVRLPSK were named phages 1, 2 and 3, respectively. The sequence data has been deposited in GenBank database and the accession numbers for phages 1–3 are JN170122, JN170123 and JN170124, respectively. Several putative motifs, such as AWxxI, RxPx or ATSHL, in the peptides were determined. The role of the identified peptides/motifs in H5N1 virus infection needs to be investigated in the future. Recently sialylgalactose-binding peptides have been selected from a phage library to develop novel drugs that interfere with the interaction between hemagglutinin (HA) of influenza virus and glycoconjugate receptors on cells [20]; Using viral hemagglutinin protein as a target and phage display technology, recombinant Fab monoclonal antibodies specific to the HA of H5N1 virus have also been characterized [8]. Nonetheless, to our knowledge, this is the first time to report the peptide sequences that can bind to the HPAI H5N1 viruses. Further experiments are needed to analyze the function of the peptides/motifs in the context of H5N1 infection.

**Table 1:** Deduced amino acid sequences of phage clones^a^.

Phage clones (number)	Phage displayed peptide sequence
Phage 1	H**AWDPI**PA**RDPF**
Phage 2	A**AWHLI**VALAPN
Phage 3	**ATSHL**HV**RLPS**K
Phage 4	H**AWDPI**PA**RDPF**
Phage 5	H**AWDPI**PA**RDPF**
Phage 6	**ATSHL**HV**RLPS**K
Phage 7	H**AWDPI**PA**RDPF**
Phage 8	**ATSHL**HV**RLPS**K
Phage 9	**ATSHL**HV**RLPS**K
Phage 10	**ATSHL**HV**RLPS**K

a Ten selected phages (phages 1 to 10) were subjected to phage DNA extraction and PCR. The deduced amino acid sequences are shown. Boldface indicates putative motifs that bind the H5N1 viruses.

### Analysis on sensitivity of phage-based ELISA

**Figure 3:**
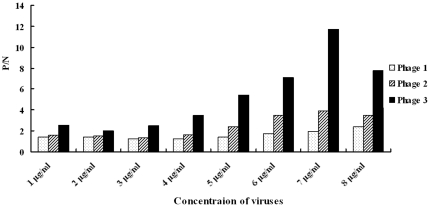
Detection limit of H5N1 viruses by phage-mediated ELISA. Serially diluted H5N1 viruses were used as coating antigens followed by successive incubation with identified phages 1 to 3, anti-M13 antibody and HRP-conjugated goat anti-rabbit antibody. The P (OD_405_ value of detected phage wells)/N (that of negative control, phage library) >2 is judged as positive results and is shown in the y axis. The experiment was performed in triplicate and the P/N value was from three independent assays. The concentration of the viruses is indicated in the x axis.

**Figure 4:**
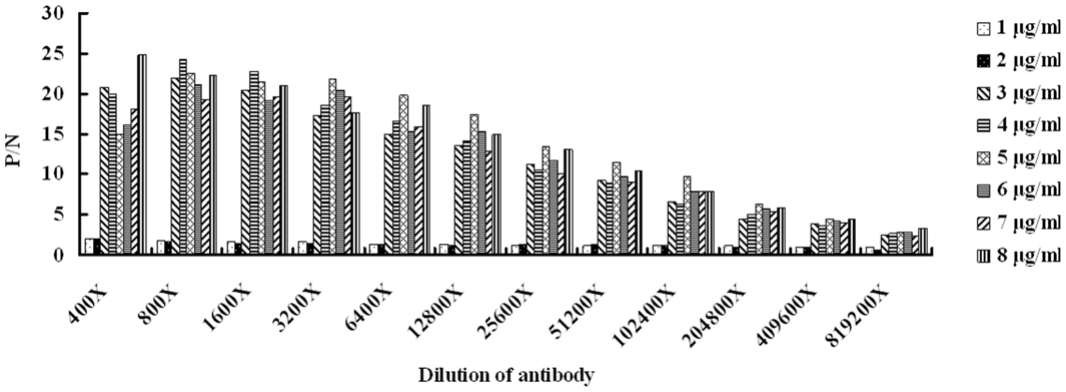
Detection limit of H5N1 viruses by antibody-based ELISA. The H5N1 viruses serially diluted in PBS was coated into ELISA plates followed by incubation of serially diluted rabbit against H5N1 serum and HRP-conjugated secondary antibody. The normal rabbit serum was used as negative control for ELISA. The P (OD_405_ value of detection wells)/N (that of negative control) >2 is judged as positive results and is shown in the y axis. The experiment was performed in triplicate and the P/N value was from three independent assays. The dilution of antibody is indicated in the x axis. The concentration of viruses is indicated.

**Figure 5:**
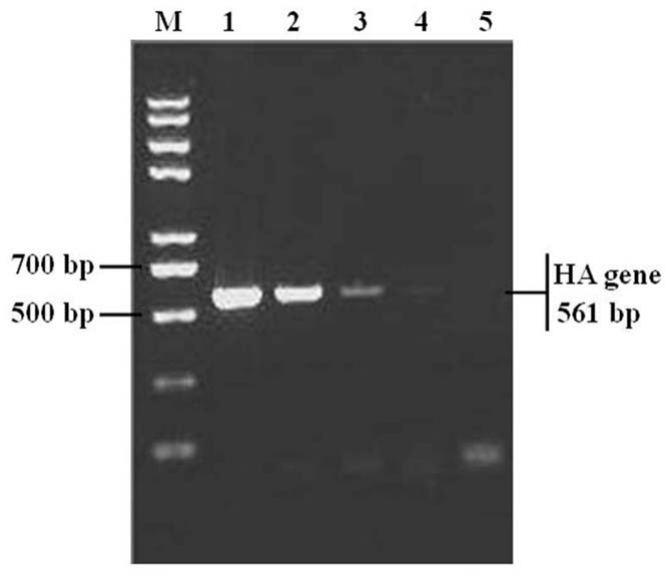
Detection limit of H5N1 viruses by RT-PCR. Viral RNA at a volume of 40 µl was extracted from 3 ml of the H5N1 viruses at a concentration of 0.647 mg/ml. Subsequently, cDNA was achieved using 5 ul extracted RNA and the resulting cDNA was ten-fold serially diluted and subjected to PCR. Lane 1: DNA marker; Lane 1, PCR product from undiluted cDNA; Lanes 2–4 were PCR products of 10-fold serially diluted cDNA. The size of amplified partial HA gene is indicated.

The sensitivity of phage-based detection was firstly analyzed by ELISA. As shown in Figure 3, the minimum quantity of the H5N1 viruses for phages 1 to 3 was 0.1 µg, 0.5 µg and 0.8 µg, respectively. The phage 1 was the most sensitive reagent used in the phage-based ELISA. Then we used the anti-H5N1 virus serum as primary antibody to analyze the sensitivity of antibody-mediated ELISA and the minimum quantity of H5N1 viruses required for the ELISA was determined as 0.3 µg (the P/N value>2) (Figure 4). Additionally, we used RT-PCR to amplify partial HA gene of the viruses. We used 1.941 mg of H5N1 viruses to extract 40 µl of RNA. Then 5 µl of the RNA was subjected to reverse transcription. The resulting cDNA was 10-fold serially diluted and used as template for PCR. As shown in Figure 5, under 1000-fold dilution, the PCR amplification of partial HA gene was positive and the minimum quantity of viruses for RT-PCR was calculated according to the equation: 1941 µg×1/8×10^−3^ = 0.243 µg. These results indicate that the phage-based ELISA is as good as if not better than antiserum-based ELISA and RT-PCR for detecting H5N1 viruses.

### Phages harboring specific peptides differentiated H5N1 viruses from other viruses

**Figure 6:**
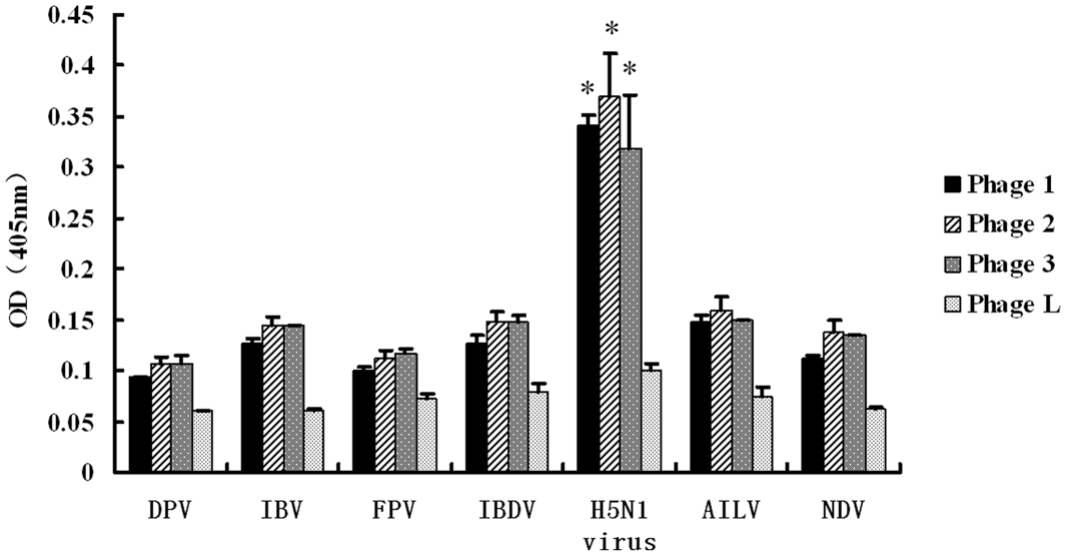
Phage-mediated ELISA for differentiating H5N1 viruses from other avian viruses. Phages (1–3) harboring specific peptides recognizing the H5N1 viruses were incubated with the duck plague virus (DPV), avian bronchitis virus (IBV), fowlpox virus (FPV), avian infectious bursal disease virus (IBDV), H5N1 virus, avian infectious laryngotracheitis virus (AILV) and newcastle disease virus (NDV). The viruses were diluted in 0.1 M NaHCO_3_ (pH8.6) to a final concentration of 15 µg/well in ELISA plates and conventional ELISA was performed as described in Materials and methods The phage complex from the phage library (Phage L) was used as control. The experiment was performed in triplicate. The name of the viruses and the OD_405_ value of individual phage are indicated in x and y axis, respectively. “*” means p<0.01 (compared with other groups).

**Figure 7:**
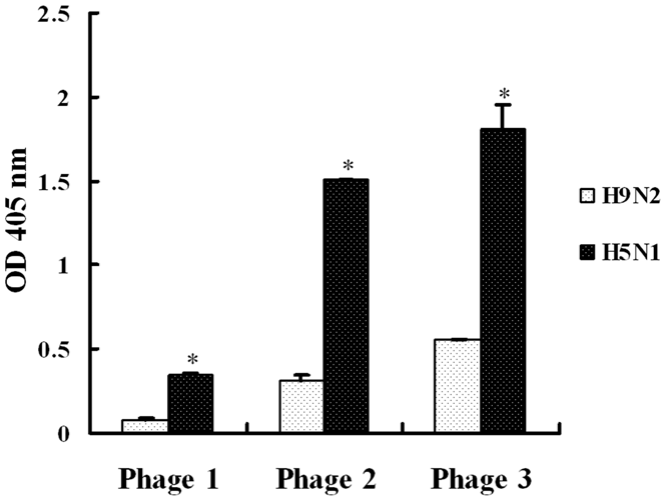
Differentiation between AIV H5N1 and H9N2 viruses by phage-mediated ELISA. The H5N1 viruses or H9N2 viruses were diluted in 0.1 M NaHCO_3_ (pH8.6) to a final concentration of 15 µg/well in ELISA plates followed by incubation with phages 1–3, anti-M13 antibody and HRP-conjugated secondary antibody. The name and OD_405_ value of individual phage are indicated in x and y axis, respectively. “*” means p<0.01 (compared with control). The experiment was performed in triplicate.

Since another purpose of this study was to develop a novel diagnostic assay to H5N1 viruses, it is clearly important to be able to distinguish influenza A virus from other viruses that might cause mixed infections. Therefore, the selected phages were analyzed for their specificities in recognizing H5N1 viruses and other avian viruses. The avian viruses selected in this study are very common in China and some of which may cause co-infection with H5N1 viruses [21]–[26]. As shown in Figure 6, the three identified phages were capable of recognizing H5N1 viruses specifically rather than other control viruses (p<0.01). At the same time, the low reactivity of the control, the phage complex from the phage library, to the targets in the ELISA excluded any artifact clearly. To further analyze the specificity of the phage-based ELISA, we included avian H9N2 influenza viruses as coating antigen and the phage-based ELISA indicated that all the three phages recognizing H5N1 viruses had lower reaction with H9N2 viruses, compared with H5N1 (Figure 7) (p<0.01). Testing against other strains of influenza A virus, or even of other influenza species such as B or C may be helpful for full evaluation of the diagnostic applications of the specific assay. Propagation of phage is relatively cheap and can be done on a large scale. Therefore, the specific phages identified in this study should be used as specific and inexpensive diagnostic reagents for detection of H5N1 viruses. Other diagnostic tests to H5N1 viruses such as real-time RT-PCR and genomic microarray assay have been reported recently [27], [28]. In the future, it would be interesting to use the phages to detect other H5N1 strains or other influenza A viruses to perform actual detection of virus from infected samples.
